# The artificial intelligence‐assisted cytology diagnostic system in large‐scale cervical cancer screening: A population‐based cohort study of 0.7 million women

**DOI:** 10.1002/cam4.3296

**Published:** 2020-07-22

**Authors:** Heling Bao, Xiaorong Sun, Yi Zhang, Baochuan Pang, Hua Li, Liang Zhou, Fengpin Wu, Dehua Cao, Jian Wang, Bojana Turic, Linhong Wang

**Affiliations:** ^1^ Department of Maternal and Child Health School of Public Health Peking University Beijing China; ^2^ National Center for Chronic and Non‐communicable Disease Control and Prevention Chinese Center for Disease Control and Prevention Beijing China; ^3^ Landing Cloud Medical Laboratory Co. Wuhan China; ^4^ Electronic and Information Engineering Department Wenhua College Wuhan China; ^5^ Landing Artificial Intelligence Center for Pathological Diagnosis Wuhan University Wuhan China

**Keywords:** artificial intelligence, cervical cancer screening, cytopathology, population‐based study

## Abstract

**Background:**

Adequate cytology is limited by insufficient cytologists in a large‐scale cervical cancer screening. We aimed to develop an artificial intelligence (AI)‐assisted cytology system in cervical cancer screening program.

**Methods:**

We conducted a perspective cohort study within a population‐based cervical cancer screening program for 0.7 million women, using a validated AI‐assisted cytology system. For comparison, cytologists examined all slides classified by AI as abnormal and a randomly selected 10% of normal slides. Each woman with slides classified as abnormal by either AI‐assisted or manual reading was diagnosed by colposcopy and biopsy. The outcomes were histologically confirmed cervical intraepithelial neoplasia grade 2 or worse (CIN2+).

**Results:**

Finally, we recruited 703 103 women, of whom 98 549 were independently screened by AI and manual reading. The overall agreement rate between AI and manual reading was 94.7% (95% confidential interval [CI], 94.5%‐94.8%), and kappa was 0.92 (0.91‐0.92). The detection rates of CIN2+ increased with the severity of cytology abnormality performed by both AI and manual reading (*P*_trend_ < 0.001). General estimated equations showed that detection of CIN2+ among women with ASC‐H or HSIL by AI were significantly higher than corresponding groups classified by cytologists (for ASC‐H: odds ratio [OR] = 1.22, 95%CI 1.11‐1.34, *P* < .001; for HSIL: OR = 1.41, 1.28‐1.55, *P* < .001). AI‐assisted cytology was 5.8% (3.0%‐8.6%) more sensitive for detection of CIN2+ than manual reading with a slight reduction in specificity.

**Conclusions:**

AI‐assisted cytology system could exclude most of normal cytology, and improve sensitivity with clinically equivalent specificity for detection of CIN2+ compared with manual cytology reading. Overall, the results support AI‐based cytology system for the primary cervical cancer screening in large‐scale population.

## Graphical abstract

This study aims to assess the role of Artificial Intelligence (AI) in the detection of early cervical cancer in a low resource setting. Our results showed that AI‐assisted cytology could identify most of negative cytology, and showed higher positive predictive value for CIN2 or worse when compared with cytologists. This study indicates that AI‐assisted cytology could be very useful tool as a primary screening method in a large‐scale cervical cancer screening program to improve its effectiveness.

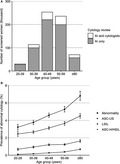

## 1 INTRODUCTION

Cervical cytology has been used for cervical cancer screening for decades, and reduced the burden of cervical cancer worldwide.^1^, ^2^, ^3^ Cytology‐based cervical cancer screening is mostly performed through microscopic observation of cervical cell morphology by cytotechnologists or cytologists.^2^, ^3^, ^4^ Cytology‐based screening strategy is recommended for population‐based cervical cancer screening in many guidelines.^5^, ^6^, ^7^ Recently, human papillomavirus (HPV) test has being recommended for cervical cancer primary screening because of a slightly higher sensitivity,^5^, ^6^, ^7^ and some countries are moving toward HPV test as primary screening or co‐testing.^8^, ^9^

Since 2009, Chinese health authorities initiated a free, population‐based cervical cancer screening program in rural areas, which screened approximately 10 million rural women per year.^10^ The initiative substantially contributed to development of cytology‐based cervical cancer screening guidelines. However, the program may not have all benefits from guidelines similar to those adopted by the western countries. There are still many challenges for the cytology‐based strategy in low‐resource settings, such as insufficient number of professionals to read the huge number of slides and lack of standardized quality control system for population‐based screening. Many women are still not screened or are under‐screened in China, and there are great disparities in cytology‐based cervical cancer screening, particularly in low‐resource settings.^11^ Therefore, decision makers are still in dilemma when they need choose the protocol for a population‐based cervical cancer screening program.

Automated cytology reading using conventional neutral network, eg the ThinPrep Imaging System and BC Focal Point GS Imaging System,^12^ has been reported as adjunct to manual cytology reading with increased sensitivity, however, the conclusions are discordant.^13^, ^14^ Recently, artificial intelligence (AI) technologies based on deep learning algorithms are developing in the field of medical diagnostics. The intelligent recognition of medical images and counting methods based on deep learning enables automatic diagnosis or tests in identifying lesions or diseases.^15^, ^16^ Previous studies showed that AI‐assisted technology might be used for segmentation of cytoplasm and identification of cervical epithelial dysplasia,^15^, ^16^, ^17^ however, the performance of AI‐assisted cytology in population‐based screening is still unclear.

In this study, we developed an AI‐assisted cytology system based on deep learning algorithms and evaluated the system in a large‐scale, population‐based cervical cancer screening program in Hubei province in China. We conducted a cohort study and assessed the effectiveness of AI‐assisted cytology compared with manual reading cytology at baseline.

## 2 MATERIAL AND METHODS

### 2.1 Study design and participants

From January 1, 2017 to December 31, 2018, we conducted a large‐scale cervical cancer screening program among women with deprived socioeconomic status in Hubei province in China, using AI‐assisted cytology system which is based on deep learning algorithms. The program enrolled women from communities in 16 cities in Hubei provinces (Figure S1), and a total of 703 103 women aged 20‐65 years participated in the program.

A real‐time subsample was randomly extracted, and double examined by cytologists and reviewed by pathologists for supervision. Then we conducted a cohort study within the program and compared the accuracy of the AI‐assisted cytology system in detection of histologically confirmed cervical intraepithelial neoplasia (CIN) or invasive cancer compared with cytology reading by cytologists at baseline.

All participants were invited to make an informed choice about participating in the cervical cancer screening. The study protocol and data retrieval were approved by the ethical committee of the National Center for Chronic and Non‐communicable Disease Control and Prevention, Chinese Center for Disease Control and Prevention (Number: NCNCD201617).

### 2.2 Artificial intelligence approach

The AI cytology system (Landing CytoScanner) was trained using a well‐defined cervical cell data set that we collected previously. Briefly, we collected 8329 cytological samples during routine cervical cancer screening program from 2012 to 2016. Samples were collected from nine different provinces. Each sample was scanned and digitalized using Olympus BX43 microscopes with digital cameras and 10× objective lens. After that, a contour‐based cell nuclei segmentation algorithm was applied to exact cell images with size of 128 × 128 centered on the nucleus centroid from slide images. The cell images were thus provided to the cytologists for classification. Each cell was classified as abnormal or normal by two cytologists from university medical centers. We selected those concordant cell images by two cytologists as training set, and excluded those with discordant classification. For the training set, there were 103 793 cell images, including 32 859 abnormal cells and 70 934 normal cells. We fed the training set into the deep learning algorithms.^17^, ^18^

The output layer was composed of three neurons, which was corresponding to normal, abnormal, and inadequate class respectively. We combined the cell nuclei segmentation algorithm and the prediction to produce the prediction score for each cell image (Figure S2). For each slide with thousands of cell images, the final prediction score was obtained by aggregating these prediction scores.

$$S=\frac{\sum_{m=1}^{M}\exp\left(‐m\right)S_{m}}{\sum_{m=1}^{M}\exp(‐m)}$$

Here, $S_{m}$ was the score of the m^th^ prediction score. The final prediction score ranged from 0 to 1, with a higher score positively associated with severe squamous intraepithelial lesions. Generally, the score of less than 0.5 was equivalent to normal cytology, whereas the score of close to 1 was more likely to be HISL or worse.

### 2.3 Procedures

**FIGURE 1:**
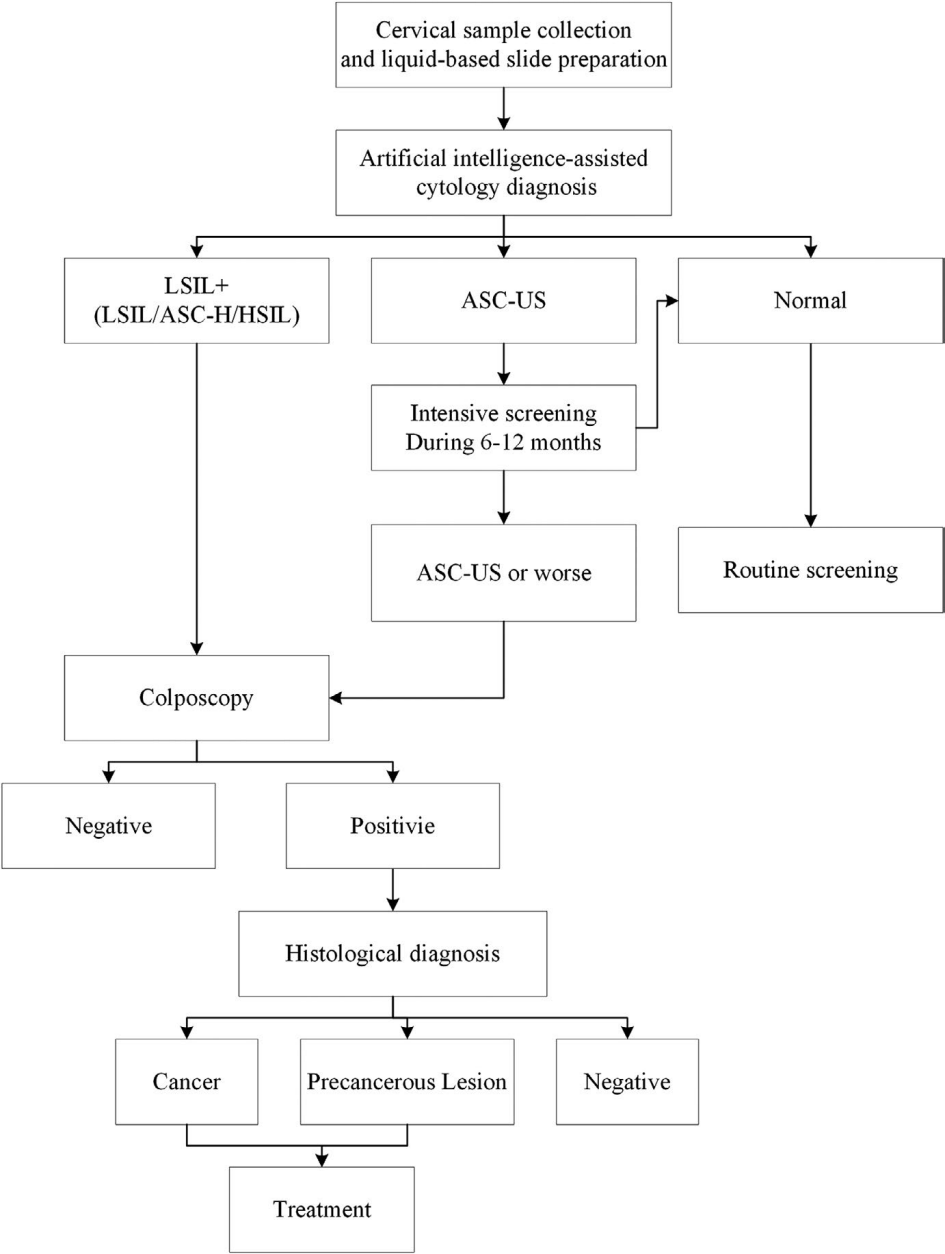
The protocol for cervical cancer screening in the program. Abbreviations: ASC‐H, atypical squamous cells, cannot rule out HSIL; ASC‐US, atypical squamous cells of undetermined significance; HSIL, high‐grade squamous intraepithelial lesion; LSIL, low‐grade squamous intraepithelial lesion

We performed the cervical cancer screening program according to the predefined protocol (Figure 1). There were 83 county or district maternal and child health care hospitals in the program, where approximately two gynecologists collected cervical samples in each hospital, using a cytology brush with a removable tip which was placed into a cytology preservation solution. Samples were sent to the Landing Medical Laboratory (Wuhan, China), and were made into slides using liquid‐based cytology method. Slides were stained with Thionin reagent Feulgen for nuclear staining, and with EA50 for cytoplasm. All slides were placed on an automatic digital pathological cell analyzer (LD DNA‐ICM II) for scanning to generate cytological images. After that, all images for each slide (including at least 5000 epithelial cells) were automatically analyzed and classified as normal, abnormal, and unsatisfactory. The system presented 20 image patches of interest with highest score, which were most likely to be abnormal, as well as final prediction scores for each slide on the digital screen. The slides with abnormal cytology classified by AI were passed to cytotechician for the Bethesda system (TBS) classification, while negative slides were given rapid review by cytotechnicians (AI arm) for rapid review. The procedure of AI‐assisted cytology was monitored and supervised by 12 cytotechnicians from Landing Medical laboratory. A panel of five independent cytologists from Landing Medical laboratory manually scanned all positive cytology slides and a randomly selected 10% of normal cytology slides under conventional microscope (manual arm). The positive slides and randomly selected negative slides were mixed, and AI‐assisted cytology results were masked to manual reading. Six independent pathologists from university medical centers rapidly reviewed these results (Table S1). The random selection of normal cytology was performed at different stages of the screening program. We defined the unsatisfactory cytology by AI system as images that included less than 5000 epithelial cells, stacked cells, slides with an obscure background, and scant cellularity. All the unsatisfactory cytology results were excluded from the comparison.

Each woman with abnormal cytology (including low‐grade squamous intraepithelial lesion [LSIL], atypical squamous cells where it was not possible to exclude high grade squamous intraepithelial lesion [ASC‐H], and high‐grade squamous intraepithelial lesion [HSIL]) identified by either AI or cytologists was referred to an immediate colposcopy and biopsy for histological confirmation. The biopsy specimens were sent to Landing Medical Laboratory for histological diagnosis and were reviewed by the panel of six independent pathologists and each biopsy was reviewed by at least two independent pathologists. Women with histologically confirmed cervical intraepithelial neoplasia grade2 (CIN2), grade3 (CIN3), and invasive cancer were sent to the hospital for immediate treatment. Women who were diagnosed as negative for intraepithelial lesion or malignancy (NILM) were recommended for a routine screening after 24 months, and women with atypical squamous cells of undetermined significance (ASC‐US) were recommended for an interim screening during 6‐12 months.

### 2.4 Statistical analysis

The estimates considering the cluster effect was used for the prevalence of abnormal cytology among screened women. The 95% confidence intervals (CI) for detection rates of CIN by abnormal cytology grades were estimated using the Fisher exact method. We tested differences in paired nominal data using McNemar's *χ* ^2^ test. For the purpose of testing the correlation between AI‐assisted cytology system and manual cytology, we tested agreement rate, and kappa overall and by different grades respectively. The cytology results were transformed to binary variables for the comparison at the threshold of ASC‐US and LSIL respectively. For multiple cytology grades, weighted kappa was used for the comparison. We also compared the positive predictive values (PPVs) between AI‐assisted cytology and cytologists, by calculating the detection rates of CIN 2+ and CIN3+ in different cytology grades. A generalized estimating equations (GEEs) with legit link were used for these data with repeated observations on each individual, adjusted for age. Odds ratios (OR) indicated the likelihood of detection of CIN2+ and CIN3+ in different cytology grades by AI‐based cytology vs those by cytologists. To calculate the sensitivity and specificity, we used histologically confirmed diagnosis as golden criteria, and selected all concordant negative women diagnosed by both AI and cytologists as negative group. Sensitivity was calculated as a number of CIN2+ cases who were classified as LSIL grade+ divided by all detected CIN2+ cases, whereas specificity was calculated from number of women who were classified as normal cytology divided by the sum of histologically confirmed negative and normal cytology classified by both AI and cytologists. All statistical analyses were done with SAS software (version 9.4) and R software ggplot package (version 3.5.4) for plot.

## 3 RESULTS

**FIGURE 2:**
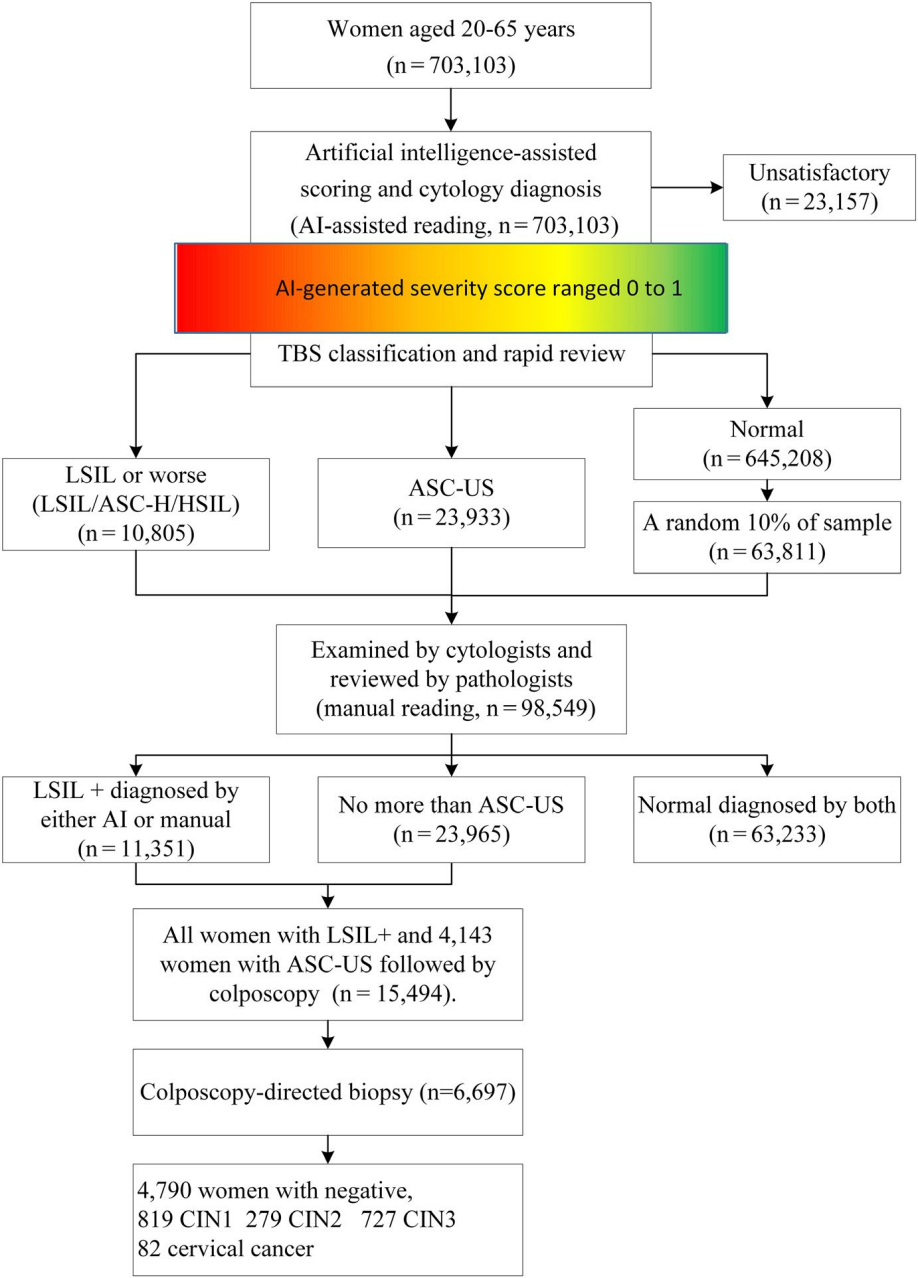
The flowchart of the AI‐assisted cervical cancer screening in study. Abbreviations: ASC‐H, atypical squamous cells, cannot rule out HSIL; ASC‐US, atypical squamous cells of undetermined significance; CIN, cervical intraepithelial neoplasia; HSIL, high‐grade squamous intraepithelial lesion; LSIL, low‐grade squamous intraepithelial lesion

**FIGURE 3:**
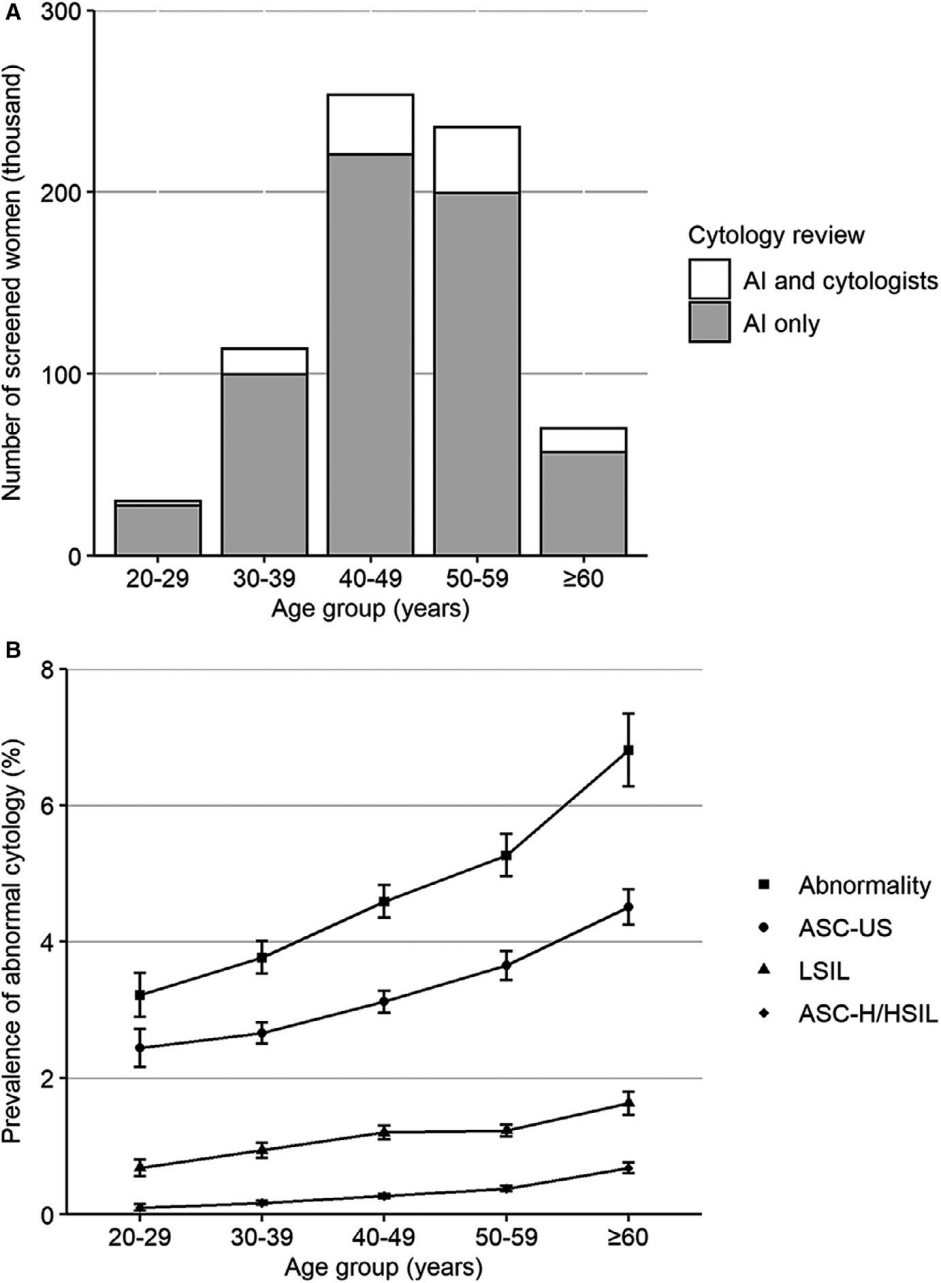
Screened women by method and prevalence of abnormal cytology. A, The number of women screened by AI only and both AI and cytologists. B, The prevalence of abnormal cytology in 703 301 women screened by AI. Abbreviations: AI, artificial intelligence; ASC‐H, atypical squamous cells, cannot rule out HSIL; ASC‐US, atypical squamous cells of undetermined significance; HSIL, high‐grade squamous intraepithelial lesion; LSIL, low‐grade squamous intraepithelial lesion. The error bar represented the 95% confidential interval

From January 1, 2017 to December 31, 2018, AI‐assisted cytology system screened 701 301 eligible women, and 15 494 women with abnormal cytology attended colposcopy (Figure 2). There were 30 035 women aged 20‐29 years, 113 970 aged 30‐39 years, 253 474 aged 40‐49 years, 235 684 aged 50‐59 years, and 69 940 aged 60 years and over (Figure 3A). The detection rate of overall abnormal cytology by AI was 4.9% (95%CI, 4.7%‐5.2%), with ASC‐US 3.4% (3.2%‐3.6%), LSIL 1.2% (1.1%‐1.3%), and ASC‐H/HSIL 0.3% (0.3%‐0.4%). The prevalence of different abnormal cytology grades substantially increased with the age group (*P*_trend_ < .001) (Figure 3B). From the eligible women, all 34 738 women with abnormal cytology and a randomly selected 63 811 of women with normal cytology classified by AI were double examined and reviewed by cytologists.

AI identified 23 157 unsatisfactory cytology (3.3%), with the main reason being less than 5000 cervical epithelial cells on the slide (94.7%).

Table 1 showed the distribution of different cytology grades between AI and manual cytology. Of all 63 811 women with normal cytology classified by AI, 63 233 (99.1%) were classified as NILM by cytologists. Among women with ASC‐US or worse (including ASC‐US, ASC‐H, LSIL, HSIL) classified by AI, 87.3% of women with ASC‐US, 85.7% of women with LSIL, 83.7% of women with ASC‐H, and 96.0% of women with HSIL were classified as the same grade by cytologists. In the cases with different diagnoses, 578 (0.9%) women with normal cytology diagnosed by AI was classified as abnormal cytology by cytologists (513 ASC‐US, 45 ASC‐H and 20 LSIL), 2569 (10.7%) women with ASC‐US diagnosed by AI were classified as normal by cytologists, whereas none of women with LSIL or worse diagnosed by AI was classified as normal by cytologists. The overall agreement rate was 94.7% (95%CI, 94.5‐94.8), and the corresponding kappa value was 0.92 (95%CI, 0.91‐0.92). The highest agreement rate was observed in the normal group (kappa = 0.93, 95%CI, 0.92‐0.93), whereas the lowest was in the ASC‐H group (kappa = 0.76, 95%CI, 0.74‐0.77).

**TABLE 1:** Comparison of AI‐assisted and manual cytology classification according to the Bethesda system

AI cytology	Manual cytology					The consistency between AI and manual cytology		
	NILM	ASC‐US	ASC‐H	LSIL	HSIL	Grade	Agreement rate % (95%CI)	Kappa (95%CI)
NILM (n = 63 811)	63 233 (99.1%)	513 (0.8%)	45 (0.1%)	20 (0.03%)	0 (0)	NILM	96.8 (96.7‐96.9)	0.93 (0.92‐0.93)
ASC‐US (n = 23 933)	2569 (10.7%)	20 883 (87.3%)	235 (1.0%)	246 (1.0%)	0 (0)	ASC‐US	95.5 (95.4‐95.6)	0.87 (0.87‐0.88)
ASC‐H (n = 1431)	0 (0)	18 (1.3%)	1227 (85.7%)	89 (6.2%)	97 (6.8%)	ASC‐H	99.2 (99.2‐99.3)	0.76 (0.74‐0.77)
LSIL (n = 8438)	0 (0)	867 (10.3%)	271 (3.2%)	7062 (83.7%)	238 (2.8%)	LSIL	98.2 (98.1‐98.3)	0.88 (0.87‐0.89)
HSIL (n = 936)	0 (0)	0 (0)	21 (2.2%)	16 (1.7%)	899 (96.0%)	HSIL	99.6 (99.6‐99.7)	0.83 (0.81‐0.84)
Total (n = 98 549)	65 802	22 281	1799	7433	1234	Total	94.7 (94.5‐94.8)	0.92 (0.91‐0.92)

Note

Data are presented as number (%).

Abbreviations: AI, artificial intelligence; NILM, negative for intraepithelial lesion or malignancy; ASC‐US, atypical squamous cells of undetermined significance; LSIL, low‐grade squamous intraepithelial lesion; ASC‐H, atypical squamous cells cannot rule out HSIL; HSIL, high‐grade squamous intraepithelial lesion; CI, confidential interval.

Table 2 showed the distribution of histologically confirmed diagnosis in both AI and cytologists. Colposcopy‐directed biopsies were performed in 6697 women with abnormal cytology diagnosed by either AI or cytologists. The biopsy identified 82 invasive cancers, 727 CIN3, 279 CIN2, and 819 CIN1. A total of 23 patients with CIN2+ (3 cancer, 13 CIN3, and 7 CIN2) were classified as normal cytology by cytologists, whereas only one CIN3 was classified as normal cytology by AI (*P* < .001). The women with CIN2, CIN3, or cancer were more likely to be classified as ASC‐US+ or LSIL+ by AI than that by cytologists.

**TABLE 2:** The distribution of histologically confirmed lesions in cytology grades by AI‐assisted and manual cytology

	Cytology grades					The difference between AI and manual cytology by histological diagnosis		
	NILM	ASC‐US	ASC‐H	LSIL	HSIL	Agreement rate (95%CI)	*P* value (≥ASC‐US)	*P* value (≥ASC‐H)
Cervicitis (n = 4790)						85.7 (84.7‐86.7)	<.001	<.001
AI	23 (0.5%)	1889 (39.4%)	330 (6.9%)	2407 (50.3%)	141 (2.9%)			
Manual	217 (4.5%)	1900 (39.7%)	387 (8.1%)	2068 (43.2%)	218 (4.6%)			
CIN1 (n = 819)						83.6 (81.1‐86.2)	NA	<.001
AI	0 (0)	104 (12.7%)	62 (7.6%)	598 (73.0%)	55 (6.7%)			
Manual	14 (1.7%)	156 (19.1%)	73 (8.9%)	503 (61.4%)	73 (8.9%)			
CIN2 (n = 279)						84.2 (80.0‐88.5)	NA	<.001
AI	0 (0)	34 (12.2%)	43 (15.4%)	171 (61.3%)	31 (11.1%)			
Manual	7 (2.5%)	54 (19.4%)	43 (15.4%)	141 (50.5%)	34 (12.2%)			
CIN3 (n = 727)						87.1 (84.6‐89.5)	<.001	<.001
AI	1 (0.1%)	65 (8.9%)	144 (19.8%)	337 (46.4%)	180 (24.8%)			
Manual	13 (1.8%)	85 (11.7%)	139 (19.1%)	303 (41.7%)	187 (25.7%)			
Cancer (n = 82)						89.0 (82.3‐95.8)	NA	.046
AI	0 (0)	8 (9.8%)	20 (24.4%)	29 (35.4%)	25 (30.5%)			
Manual	3 (3.7%)	9 (11.0%)	18 (22.0%)	25 (30.5%)	27 (32.9%)			

Abbreviations: AI, artificial intelligence; ASC‐H, atypical squamous cells, cannot rule out HSIL; ASC‐US, atypical squamous cells of undetermined significance; CI, confidential interval; CIN1, CIN2, or CIN3, cervical intraepithelial neoplasia grade 1, 2, or 3; HSIL, high‐grade squamous intraepithelial lesion; LSIL, low‐grade squamous intraepithelial lesion; NILM, negative for intraepithelial lesion or malignancy.

**FIGURE 4:**
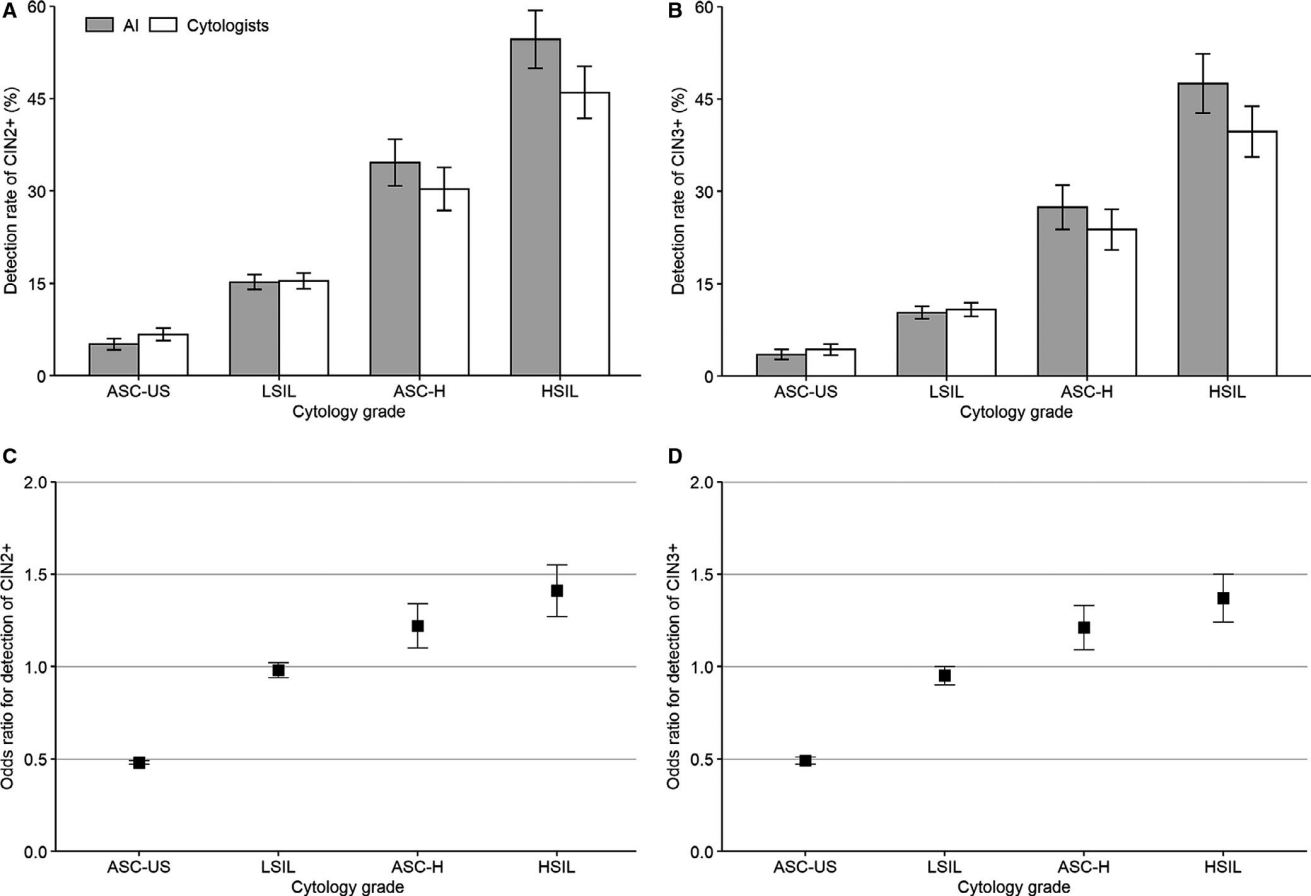
Detection of CIN2+ and CIN3+ in cytology grades between AI‐assisted and manual cytology. A, Detection of CIN2+ in AI‐assisted cytology and manual cytology. B, Detection of CIN3+ in AI assisted cytology and manual cytology. C, Odds ratio of AI relative to manual cytology for detection of CIN2+. D, Odds ratio of AI relative to manual cytology for detection of CIN3+. Abbreviations: AI, artificial intelligence; ASC‐H, atypical squamous cells, cannot rule out HSIL; ASC‐US, atypical squamous cells of undetermined significance; CI, confidential interval; CIN, cervical intraepithelial neoplasia; HSIL, high‐grade squamous intraepithelial lesion; LSIL, low‐grade squamous intraepithelial lesion; CIN2+, cervical intraepithelial neoplasia grade 2 or worse; CIN3+, cervical intraepithelial neoplasia grade 3 or worse. The error bar represented the 95% confidential interval

Figure 4 and Table S3 showed the detection of CIN2+ or CIN3+ among women who were classified with different cytology grades by either AI or cytologists. In the AI group, the detection of CIN2+ among women with ASC‐US, LSIL, ASC‐H, and HSIL was 5.1% (95%CI, 4.2%‐6.0%), 15.2% (14.0%‐16.3%), 34.6% (30.8%‐38.4%), and 54.6% (49.9%‐59.3%), respectively, with significantly increasing trend (*P*_trend_ < .001). The detection for CIN3+ showed a similar pattern. Age‐adjusted GEEs showed that the detection of CIN2+ or CIN3+ among women with ASC‐US classified by AI were significantly less than corresponding grade classified by cytologists (OR = 0.48, 95%CI 0.47‐0.49 for CIN2+, and 0.49, 95%CI 0.47‐0.50 for CIN3+). In the LSIL grade, detection of CIN2+ and CIN3+ were similar between AI and cytologists, however, detection of CIN2+ and CIN3+ were significantly higher among women with ASC‐H and HSIL classified by AI than those with corresponding grades classified by cytologists (*P* < .001 for all).

Sensitivity and specificity for detection of histologically confirmed lesions or cancer in AI and cytologists were calculated among women with concordant normal cytology and those diagnosed by biopsy (Table 3).

**TABLE 3:** Sensitivity and specificity of AI‐assisted and manual cytology for detection of histologically confirmed cervical lesions^a^

	Sensitivity (95%CI)				Specificity (95%CI)			
	AI	Manual	Difference	*P* value	AI	Manual	Difference	*P* value
CIN1+	88.9 (87.5‐90.3)	82.1 (80.4‐83.8)	6.8 (4.5‐9.0)	<.001	95.8 (95.6‐95.9)	96.1 (95.9‐96.2)	−0.3 (−0.5 to −0.1)	<.001
CIN2+	90.1 (88.3‐91.9)	84.3 (82.1‐86.4)	5.8 (3.0‐8.6)	<.001	94.8 (94.6‐94.9)	95.2 (95.0‐95.3)	−0.4 (−0.2 to −0.6)	<.001
CIN3+	90.9 (88.9‐92.8)	86.4 (84.0‐88.8)	4.5 (1.4‐7.5)	<.001	94.4 (94.3‐94.6)	94.9 (94.7‐95.0)	−0.4 (−0.7 to −0.2)	<.001

Abbreviations: AI, artificial intelligence; CI, confidential interval; CIN, cervical intraepithelial neoplasia.

^a^ A total of 69 906 cases for sensitivity and specificity analyses, including 1907 CIN 1 to 3 grades, 4790 negative, and 63 209 women with normal cytology classified by both AI and manual cytology.

At the threshold of LSIL+, AI achieved substantially higher sensitivity for CIN2+ (difference = 5.8%, 95%CI 3.0%‐8.6%) and CIN3+ (difference = 4.5%, 95%CI 1.4%‐7.5%) than manual cytology, respectively, whereas corresponding specificity had a slight decrease compared with cytologists (difference 0.4%).

## 4 DISCUSSION

Our study among 0.7 million women yields several novel findings about the role of AI‐assisted cytology in population‐based cervical cancer screening. First, cytologists confirmed more than 99% of women classified as normal by AI, and the agreement rate for normal cytology between AI and manual cytology was at 97%. Second, AI‐assisted cytology showed higher sensitivity with inconsequential decrease in specificity for the detection of CIN2+ compared with a manual cytology reading. Within LSIL grade, the detection of CIN2+ and CIN3+ was equivalent between AI arm and manual arm; thereafter, the detection of CIN2+ was 20% higher in ASC‐H grade and 40% higher in HSIL grade classified by AI when compared to manual reading. These findings indicate that the AI‐assisted cytology system could reduce the number of negative cytology slides for manual reading and increase the efficiency in detection of CIN2+ in population‐based screening.

Our study showed high agreement rate for normal cytology grade between AI and manual reading, which were also reported with the automated ThinPrep imager.^19^, ^20^, ^21^ More than 99% of women classified as normal cytology by AI were confirmed by manual reading, suggesting that most of the women with normal cytology could be primarily excluded by AI. The false negative of manual cytology reading is correlated with the low prevalence of abnormal cytology,^22^ whereas prevalence of the abnormal cytology is approximately 3%‐5% in general population.^23^, ^24^, ^25^ Similar to the FocalPoint system classifying 25% of slides as needing no further review,^12^ our system designates majority of slides most likely to be normal as only needing rapid review, indirectly increasing focus on the positive cytology slides. Indeed, the detection rate of abnormal cytology in our study is closed to 5%, being 20% higher than national organized cervical cancer screening program (3.2% for TBS report),^26^ and higher than some developed countries.^24^, ^25^

AI‐assisted cytology showed increased sensitivity with inconsequential decrease in specificity for detection of CIN2+, compared with manual reading, in accordance with previously published studies using automated cytology,^20^, ^21^ but inconsistent with MAVARIC trial.^13^ Although the well‐designed trial showed that it is less sensitive for automated‐assisted cytology than manual reading,^13^ different comparison may exist under the distinct context of population size, performance of manual liquid‐based cytology, and AI algorithms. In the present study, the detection of histological CIN2+ among women classified as normal by manual reading and abnormal by AI, was substantially higher than that among women classified as normal by AI and abnormal by manual reading. Compared with the procedure of ThinPrep imager or BD FocalPoint system, the scanner provides 20 image patches (usually containing exfoliated cells) most likely to be abnormal, as well as a prediction score, indicating the likelihood of severity of the disease. This may increase the focus of cytoscreener on positive slides. The detection of CIN2+ in our study is higher than that published for a national program (155 vs 125 per 100 000),^26^ however, most ASC‐US are not referred to an immediate colposcopy that would underestimate the performance of AI.

An important issue of cytology‐based cervical cancer screening is the management of ASC‐US, in which risk of high‐grade lesions or cancer varies greatly.^27^, ^28^ Inappropriate triage may result in an over referral to colposcopy, or a delayed diagnosis and treatment.^29^ Although human papillomavirus test, genotyping or some biomarkers (eg methylation, p16/Ki‐67) provide technology for triaging ASC‐US, these algorithms are limited or not available in a low‐resource settings.^29^, ^30^ In our study, we adopted the protocol that deferred women with ASC‐US to an intensive screening at the interval of 6‐12 months rather than an immediate colposcopy. Interestingly, the AI system seems to reclassify more CIN2+ cases in ASC‐US grade classified by manual cytology to ASC‐H or LSIL. The results support the decision of delaying immediate colposcopy in the ASC‐US group and decrease repeated screening for those women with ASC‐US. Nonetheless, the risk of CIN2+ in women with ASC‐US at intervals needs to be evaluated in the continuous screening.

Samples of discordant pairs associated with undelaying CIN2+(23 samples) were reviewed by two cytologists independently. 15 (65%) cases were diagnosed as normal cytology by two cytologists, however, in remaining cases, 8 cases were diagnosed as ASC‐US or worse by at least one cytologist. 22 of these cases were diagnosed as ASC‐US by AI but with less than three metaplastic squamous cells presented in each slide. These findings show that scanty abnormal material is difficult to identify in the case of manual cytology reading, which has been reported by Halford and colleagues.^31^ Workloads (>35 slides/ day) were also reported^32^ and could have affected the vigilance of manual reading. Although prior study doubted that the auto‐location may neglect abnormal cells at the periphery of the segmented fields of view,^13^ such cells could be identified by splicing image patches related to one cell in our AI algorithm.

Cytology has many advantages (eg practicability, simplicity, sufficient evidence in reducing cervical cancer burden), however, cytology‐based screening strategy requires high quality of health system, including sample collection and preparation, skilled professionals, and strict quality control system in the laboratory. Liquid‐based technology facilitates better preparation of samples and reduces the unsatisfactory rate,^14^, ^33^ however, it has a few effects on the detection of precancerous lesions or cancer compared with conventional cytology.^4^, ^14^, ^33^ Furthermore, there are great heterogeneities in cytology classification within different cytologist’ groups.^34^ AI‐assisted cytology system provides opportunities to address these difficulties,^14^, ^35^ for example, inexhaustible scanning of the slide image, constant vigilance for abnormal cells, repeatable cytology diagnosis, and quantitative analysis of the severity of cases, which aid cytologists or cytotechnologists in screening cervical dysplastic cells with more accuracy. AI‐assisted cytology system uses liquid based slides, which enables HPV test triage by using residual samples. Additionally, cytologists could remotely review the cytology classification through the network to address the inequalities of health resources across geographic areas.

Although the performance of automated‐assisted cytology reading as primary screening was reported previously,^36^, ^37^ to our best knowledge, our study was the largest population‐based cervical cancer screening using AI‐assisted cytology reading in the low‐ and middle‐income countries. Besides, there are some ongoing cervical cancer screening programs using AI‐assisted cytology systems supported by the government in other provinces in China, such as Yunnan, Shanxi, and Fujian, covering more than 400 000 eligible women. This model is being proved to be practical in China and can be reproducible in other developing countries. Moreover, technological advancements and data accumulation might enable the AI system to be more intelligent and used more generally.

There are several limitations in our study. First, only 6697 women with abnormal cytology were verified by colposcopy‐directed biopsy and histological confirmation. This was mainly the result of our screening protocol because women with ASC‐US were deferred to an intensive screening during 6‐12 months to reduce the over referral of colposcopy. However, this may not have any effect on our results because the proportions of women who were referred to an immediate colposcopy were not significantly different between AI and manual reading. Nonetheless, more data about histology confirmation and incident rounds of screening is needed for the evaluation of ASC‐US group progression to CIN2+. The unsatisfactory rate of AI‐assisted cytology system compared with manual cytology was not thoroughly evaluated due to the exclusion of these unsatisfactory cases from manual cytology. The issues and solutions of unsatisfactory in AI‐assisted cytology need further study.

In conclusion, AI‐assisted cytology could distinguish most of normal cytology, and improve sensitivity for detection of CIN2+ with clinically equivalent specificity compared with manual cytology reading. This study indicates that AI‐assisted cytology system could be used as primary screening to improve the accuracy and efficiency of cytology in population‐based cervical cancer screening.

## 5 ETHICS COMMITTEE APPROVAL

The study protocol was approved by the ethical review committee of the National Center for Chronic and Non‐communicable Disease Control and Prevention, China CDC (Number: NCNCD201617). Written informed consent was obtained from all study participants.

## CONFLICT OF INTEREST

The authors made no disclosures.

## AUTHOR CONTRIBUTIONS

Xiaorong Sun, Linhong Wang, and Bojana Turic contributed to the study design, planning, organization, and revision. Heling Bao contributed to the study design, organization, data analyses, and article drafting. Yi Zhang and Baochuan Pang contributed to the development and train of artificial intelligence mode and device supply. Hua Li, Liang Zhou, and Fengpin Wu contributed to the cytology examination and diagnosis. Jian Wang and Dehua Cao contributed to the data collection and article revision.

## Supporting information

Appendix

Figure S1

Figure S2
