# Supplementary material for: The artificial intelligence‐assisted cytology diagnostic system in large‐scale cervical cancer screening: A population‐based cohort study of 0.7 million women
Source: Cancer Med. 2020 Jul 22;9(18):6896–906. doi: 10.1002/cam4.3296 (PMC7520355; doi:10.1002/cam4.3296)
Supplement: Supplementary file 1 — Appendix [file CAM4-9-6896-s001.docx]

**Supplementary Appendix**

This appendix has been provided by the authors to give readers additional information about their work.

Supplement to: Bao H, Sun X, Zhang Y, Pang B, Li H, Zhou L, Wu F, Cao D, Wang J, Bojana T, Wang L. The artificial intelligence-assisted cytology diagnostic system in large-scale cervical cancer screening: a population-based cohort study of 0.7 million women.

**
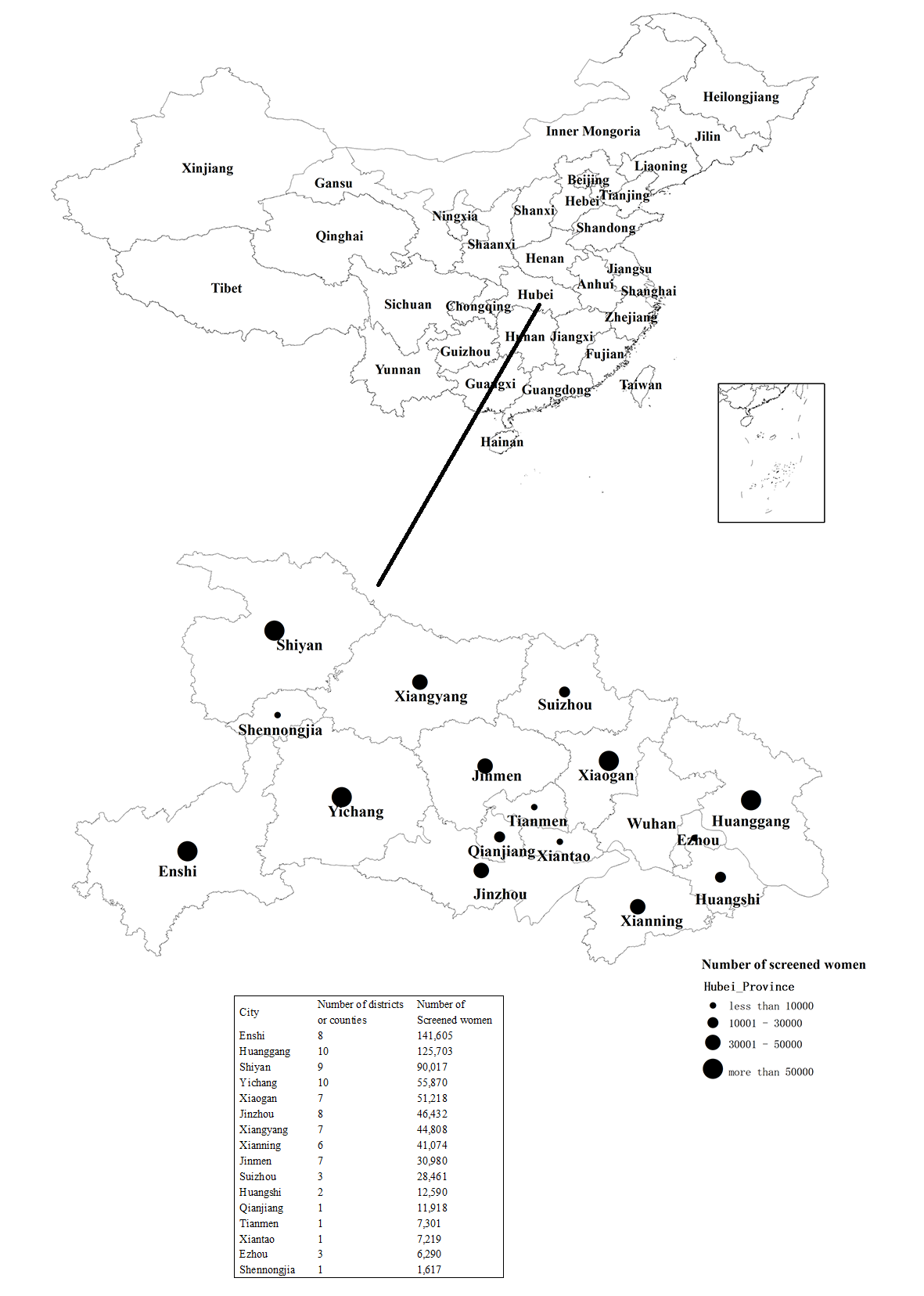
**

**eFigure 1**. The distribution of 703,103 screened women in Hubei province, China.


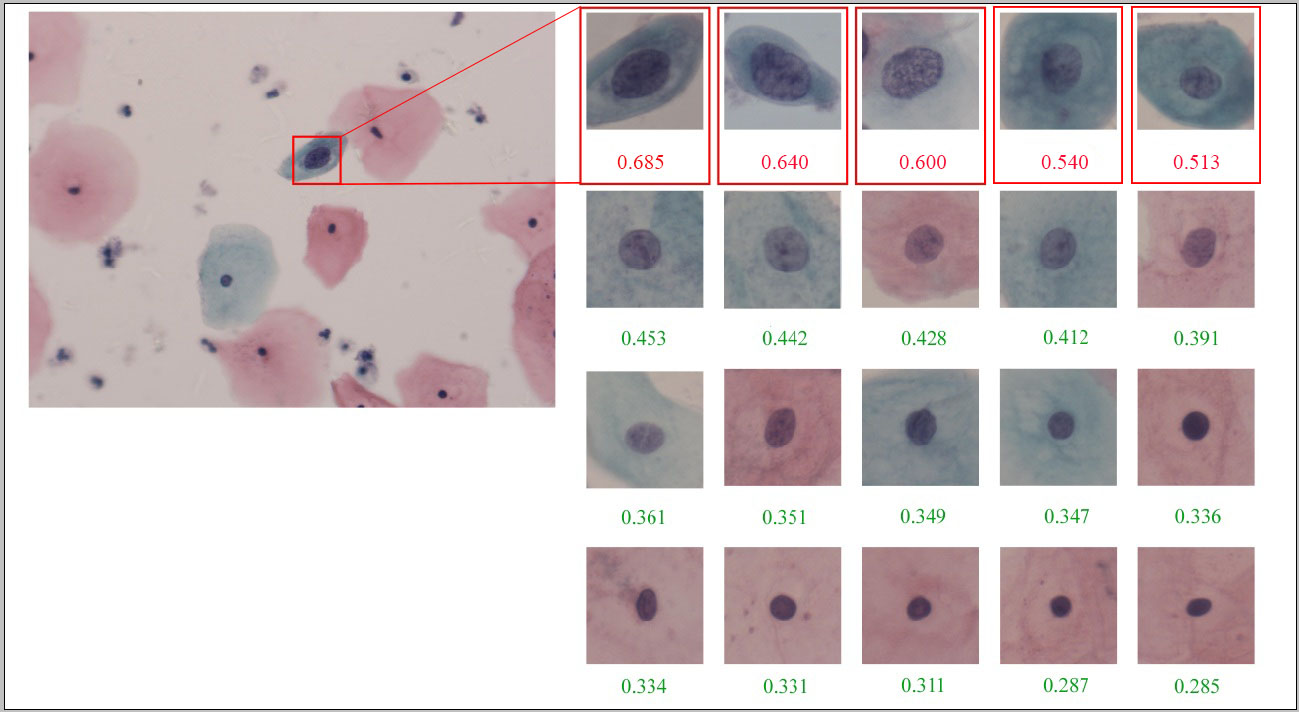


**eFigure 2:** The electronic images of suspicious cervical cells detected by AI-assisted cytology system with score.

**eTABLE 1.** The Panel of Pathologists, Cytologists, and Cytotechnologists in the Study

| **Group** | **Name** | **Title** | **Institution** | **Duty in the program** |
| --- | --- | --- | --- | --- |
| Pathologists | DY | Professor | University or public medical center | Review in diagnosis of cytology and histopathology |
| Pathologists | CS | Professor | University or public medical center | Review in diagnosis of cytology and histopathology |
| Pathologists | LH | Professor | University or public medical center | Review in diagnosis of cytology and histopathology |
| Pathologists | WGP | Professor | University or public medical center | Review in diagnosis of cytology and histopathology |
| Pathologists | TL | Professor | University or public medical center | Review in diagnosis of cytology and histopathology |
| Pathologists | WXB | Professor | University or public medical center | Review in diagnosis of cytology and histopathology |
| Cytologists | DL | Attending doctor | Landing laboratory | Cytology diagnosis |
| Cytologists | LH | Physician | Landing laboratory | Cytology diagnosis |
| Cytologists | WFP | Physician | Landing laboratory | Cytology diagnosis |
| Cytologists | XWY | Assistant physician | Landing laboratory | Cytology diagnosis |
| Cytologists | ZY | Assistant physician | Landing laboratory | Cytology diagnosis |
| Cytotechnologist | HFS | Chief technician | Landing laboratory | AI-assisted cytology diagnosis |
| Cytotechnologist | XJ | Competent technician | Landing laboratory | AI-assisted cytology diagnosis |
| Cytotechnologist | NM | Technician | Landing laboratory | AI-assisted cytology diagnosis |
| Cytotechnologist | LB | Technician | Landing laboratory | AI-assisted cytology diagnosis |
| Cytotechnologist | JT | Technician | Landing laboratory | AI-assisted cytology diagnosis |
| Cytotechnologist | HT | Technician | Landing laboratory | AI-assisted cytology diagnosis |
| Cytotechnologist | QHJ | Technician | Landing laboratory | AI-assisted cytology diagnosis |
| Cytotechnologist | CY | Technician | Landing laboratory | AI-assisted cytology diagnosis |
| Cytotechnologist | YF | Technician | Landing laboratory | AI-assisted cytology diagnosis |
| Cytotechnologist | LXM | Technician | Landing laboratory | AI-assisted cytology diagnosis |
| Cytotechnologist | WSY | Technician | Landing laboratory | AI-assisted cytology diagnosis |
| Cytotechnologist | ZJY | Technician | Landing laboratory | AI-assisted cytology diagnosis |

**eTABLE 2.** Characteristics of Screened Women in the Study

| **Characteristics** | **N (%)** |
| --- | --- |
| **Total** | 703,103 |
| **Age group** |  |
| 20-29 | 30,035 (4.3) |
| 30-39 | 113,970 (16.2) |
| 40-49 | 253,474 (36.1) |
| 50-59 | 235,684 (33.5) |
| More than 60 | 69,940 (9.9) |
| **AI-assisted cytology** |  |
| NILM | 645,208 (91.8) |
| ASC-US | 23,933 (3.4) |
| LSIL | 8,438 (1.2) |
| ASC-H | 1,431 (0.2) |
| HSIL | 936 (0.1) |
| Unsatisfactory | 23,157 (3.3) |
| Inadequate cervical epithelial cells (<5,000) | 21,930 (94.7) |
| Stacked cell | 741 (3.2) |
| Unclear background | 255 (1.1) |
| Unclear cell | 231 (1.0) |
| **A randomly selected 10% of negative samples** |  |
| 20-29 | 1,862 (2.9) |
| 30-39 | 10,080 (15.8) |
| 40-49 | 21,673 (34.0) |
| 50-59 | 22,884 (35.9) |
| More than 60 | 7,312 (11.5) |
| **The sub-sample for manual cytology** |  |
| 20-29 | 2,523 (2.6) |
| 30-39 | 14,193 (14.4) |
| 40-49 | 32,785 (33.3) |
| 50-59 | 36,197 (36.7) |
| More than 60 | 12,851 (13.0) |

Note: Data are presented as number (%).

Abbreviations: IQR=inter-quartile range. AI=artificial intelligence. NILM=negative for intraepithelial lesion or malignancy. ASC-US=atypical squamous cells of undetermined significance. LSIL=low-grade squamous intraepithelial lesion. ASC-H=atypical squamous cells, cannot rule out HSIL. HSIL=high-grade squamous intraepithelial lesion.

**eTABLE 3.** The Difference in Detection of Histologically Confirmed CIN2 or Worse between AI-assisted and Manual Cytology.

| **TBS grade** | **Histopathologic diagnosis** | | |  | **Odds ratio (95%CI) for detection CIN2 or worse, p value** | **Odds ratio (95%CI) for CIN3 or worse, p value** |
| --- | --- | --- | --- | --- | --- | --- |
|  | **Rate of Cervicitis**  **% (95%CI)** | **Rate of CIN1,**  **% (95%CI)** | **Rate of CIN2 or worse, % (95%CI)** | **Rate of CIN3 or worse, % (95%CI)** |  |  |
| **ASC-US** |  |  |  |  |  |  |
| AI (n=2100) | 90.0 (88.7-91.2) | 5.0 (4.0-5.9) | 5.1 (4.2-6.0) | 3.5 (2.7-4.3) | 0.48 (0.47-0.49) | 0.49 (0.47-0.50) |
| Manual (n=2204) | 86.2 (84.8-87.7) | 7.1 (6.0-8.2) | 6.7 (5.7-7.8) | 4.3 (3.4-5.1) | p<0.001 | p=0.008 |
| **LSIL** |  |  |  |  |  |  |
| AI (n=3542) | 68.0 (66.4-69.5) | 16.9 (15.7-18.1) | 15.2 (14.0-16.3) | 10.3 (9.3-11.3) | 0.98 (0.94-1.02) | 0.95 (0.91-1.00) |
| Manual (n=3040) | 68.0 (66.4-69.7) | 16.6 (15.2-17.9) | 15.4 (14.1-16.7) | 10.8 (9.7-11.9) | p=0.341 | p=0.056 |
| **ASC-H** |  |  |  |  |  |  |
| AI (n=599) | 55.1 (51.1-59.1) | 10.4 (7.9-12.8) | 34.6 (30.8-38.4) | 27.4 (23.8-31.0) | 1.22 (1.11-1.34) | 1.21 (1.09-1.33) |
| Manual (n=660) | 58.6 (54.9-62.4) | 11.1 (8.7-13.5) | 30.3 (26.8-33.8) | 23.8 (20.5-27.0) | p<0.001 | P<0.001 |
| **HSIL** |  |  |  |  |  |  |
| AI (n=432) | 32.6 (28.2-37.1) | 12.7 (9.6-15.9) | 54.6 (49.9-59.3) | 47.5 (42.7-52.2) | 1.41 (1.28-1.55) | 1.37 (1.25-1.50) |
| Manual (n=539) | 40.5 (36.3-44.6) | 13.5 (10.7-16.4) | 46.0 (41.8-50.2) | 39.7 (35.6-43.8) | p<0.001 | p<0.001 |

Abbreviations: AI=artificial intelligence. NILM=negative for intraepithelial lesion or malignancy. ASC-US=atypical squamous cells of undetermined significance. LSIL=low-grade squamous intraepithelial lesion. ASC-H=atypical squamous cells, cannot rule out HSIL. HSIL=high-grade squamous intraepithelial lesion. CIN1, CIN2, or CIN3=cervical intraepithelial neoplasia grade 1, 2, or 3. CI=confidential interval.
