# Supplementary figures and images for: The artificial intelligence‐assisted cytology diagnostic system in large‐scale cervical cancer screening: A population‐based cohort study of 0.7 million women
Source: Cancer Med. 2020 Jul 22;9(18):6896–906. doi: 10.1002/cam4.3296 (PMC7520355; doi:10.1002/cam4.3296)

**
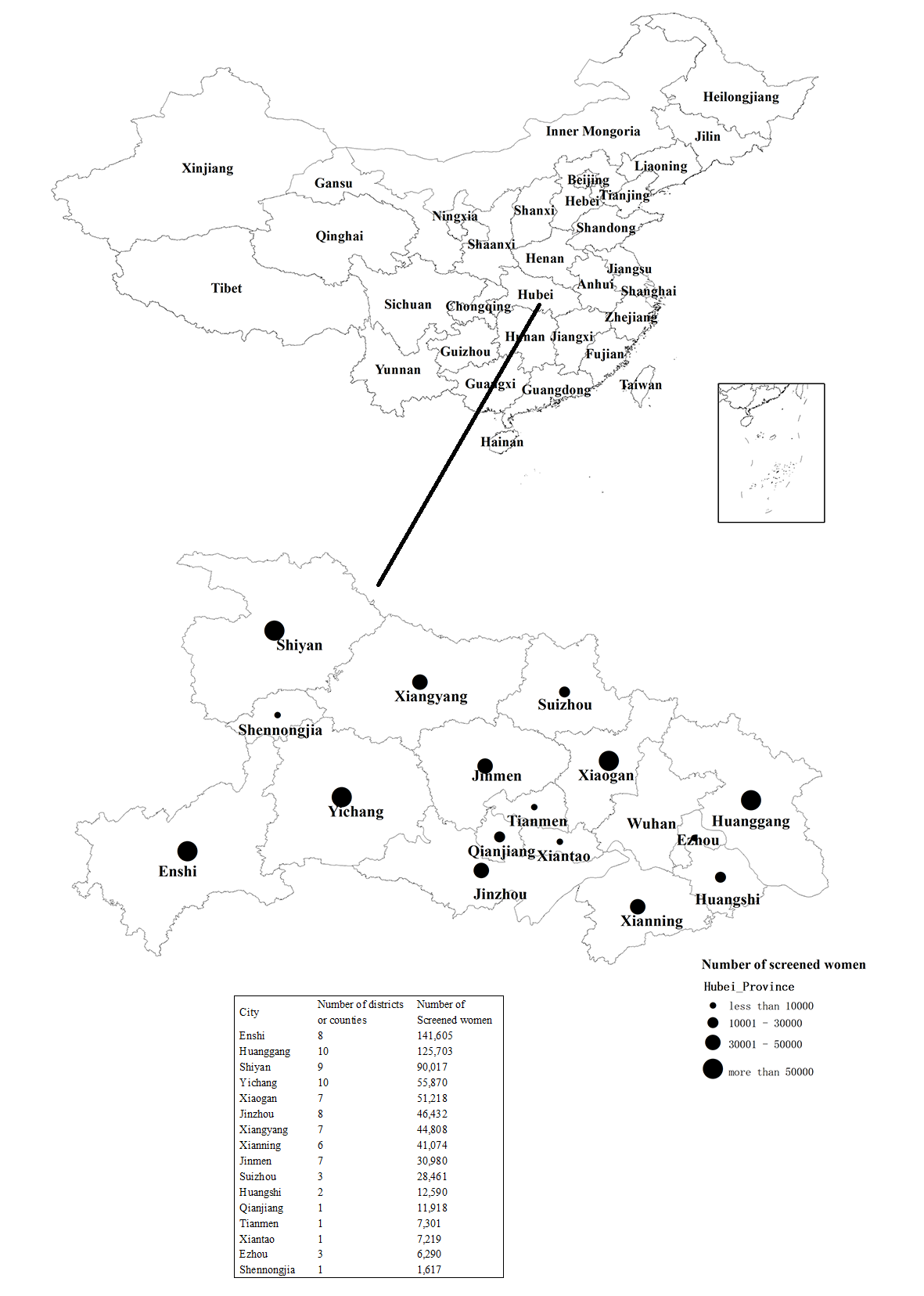
**

Supplement: Supplementary file 2 — Figure S1 [file CAM4-9-6896-s002.docx]
