# Supplementary material for: The artificial intelligence‐assisted cytology diagnostic system in large‐scale cervical cancer screening: A population‐based cohort study of 0.7 million women
Source: Cancer Med. 2020 Jul 22;9(18):6896–906. doi: 10.1002/cam4.3296 (PMC7520355; doi:10.1002/cam4.3296)
Supplement: Supplementary file 3 — Figure S2 [file CAM4-9-6896-s003.docx]

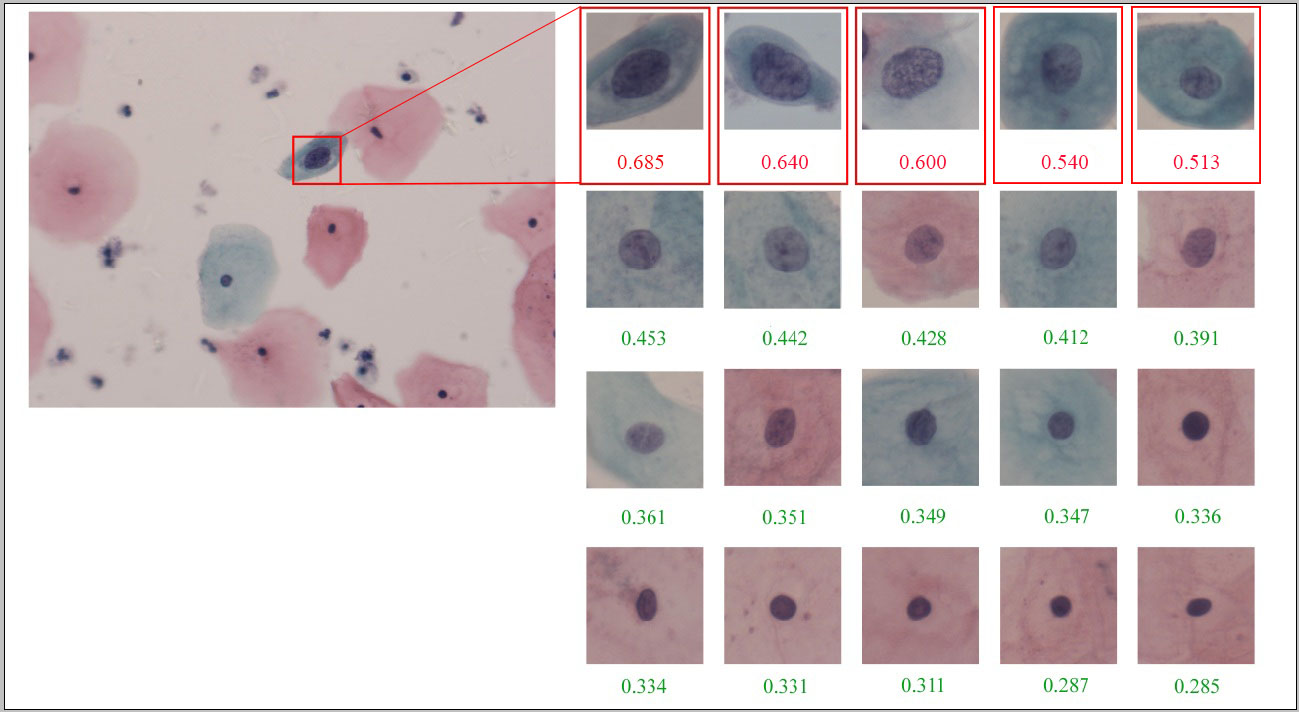


**eFigure 2:** The electronic images of suspicious cervical cells detected by AI-assisted cytology system with score.
